# *TITF1* Screening in Human Congenital Diaphragmatic Hernia (CDH)

**DOI:** 10.3390/children9081108

**Published:** 2022-07-23

**Authors:** Maria Eugenia Gulino, Giuseppe Martucciello, Elio Biffali, Patrizia Morbini, Roberta Patti, Marco Borra, Maria Grazia Scuderi

**Affiliations:** 1 UOC di Chirurgia Pediatrica, Fondazione IRCCS Policlinico San Matteo, 27100 Pavia, Italy; 2 Pediatric Surgery DiNOGMI, University of Genova, 16147 Genoa, Italy; martucciello@yahoo.com; 3 Department of Paediatric Surgery, IRCCS Giannina Gaslini Children’s Hospital, 16147 Genoa, Italy; 4 Stazione Zoologica “A. Dohrn”, 80121 Naples, Italy; elio.biffali@szn.it; 5 UOC Anatomia Patologica, Fondazione IRCCS Policlinico San Matteo, 27100 Pavia, Italy; patrizia.morbini@unipv.it; 6 UOC di Chirurgia Pediatrica, Ospedale Policlinico San Marco, 95121 Catania, Italy; roberta.patti92@gmail.com (R.P.); mgscuderi@gmail.com (M.G.S.)

**Keywords:** congenital diaphragmatic hernia (CDH), pulmonary hypoplasia (PH), molecular genetics, histochemistry, *TITF1*

## Abstract

TITF1 (Thyroid Transcription Factor-1) is a homeodomain-containing transcription factor. Previous studies showed that *Titf1* null mice are characterized by failure of tracheo-oesophageal separation and impaired lung morphogenesis resulting in Pulmonary Hypoplasia (PH). In this study, we aim to evaluate the role of *TITF1* in the pathogenesis of congenital diaphragmatic hernia (CDH) in humans. We investigated *TITF1* expression in human trachea and lungs and performed direct mutation analysis in a CDH population. We studied 13 human fetuses at 14 to 24 weeks of gestation. Five μm sections were fixed in paraformaldehyde and incubated with anti-TITF1 primary antibody. Positive staining was visualized by biotinylated secondary antibody. We also performed *TITF1* screening on genomic DNA extracted from peripheral blood of 16 patients affected by CDH and different degrees of PH, searching for mutations, insertions, and/or deletions, by sequencing the exonic regions of the gene. Histochemical studies showed positive brown staining of fetal follicular thyroid epithelium, normal fetal trachea, and normal fetal lung bronchial epithelium. Fetal esophageal wall was immunohistochemically negative. Molecular genetic analysis showed complete identity between the sequences obtained and the Wild Type (WT) form of the gene in all cases. No mutation, insertion and/or deletion was detected. Although *TITF1* is expressed in the human fetal lung and has been considered to have a role in the pathogenesis of PH in CDH, the results of our study do not support the hypothesis that *TITF1* mutations play a key role in the etiopathogenesis of CDH.

## 1. Introduction

Congenital Diaphragmatic Hernia (CDH) is a developmental defect characterized by the partial or complete absence of the diaphragm, the muscle that separates the chest cavity from the abdominal cavity, followed by the herniation of the abdominal organs into the thorax. CDH can occur as an isolated defect, in combination with multiple congenital anomalies, or as part of a well-defined syndrome [1]. According to the current literature, the incidence of CDH ranges from 0.8–5/10,000 newborns and varies across the population [2,3,4,5]. This defect is usually associated with bilateral Pulmonary Hypoplasia (PH), characterized by a reduction in the airway branching with smaller airspaces and pulmonary vascular abnormalities including a reduced number of vascular branches and thicker pulmonary arterial walls [6]. The hypermuscularization of the pulmonary bed clinically translates into pulmonary hypertension. In addition, an inadequate surfactant production is present [7]. Over time, the improvement of prenatal assessment, antenatal interventions, and postnatal management have allowed to improve the survival rate in CDH [8], yet the mortality rate remains high for patients affected by pulmonary complications related to pulmonary hypoplasia and persistent pulmonary hypertension [9,10]. Therefore, prevention of lung maldevelopment in CDH remains a priority. To this end, the comprehension of the underlying causes of PH is critical.

Although PH in CDH was initially thought to be merely secondary to the diaphragmatic defect [11,12], its embryologic origins are still not clearly understood. More recent studies suggest that PH is not determined by the mass effect caused by the herniation of abdominal viscera in the chest [13,14,15] and that it can be associated with—rather than secondary to—diaphragmatic defects [16,17].

A genetic relation is found in 30% of CDH cases. Within this group, 1–2% are familial, while most of the cases are sporadic [18], leading to the hypothesis that de novo variants are an important etiological mechanism [19]. Because of its complexity, CDH is assumed to be a multifactorial disease involving genetic, environmental, and dietary variables [18,20,21].

Among the possible candidate genes for a predisposing role in CDH is *TITF1* (Thyroid Transcription Factor-1) [16]. TITF1) is a homeodomain-containing transcription factor that was first identified as a nuclear protein able to bind to specific DNA sequences present in the thyroglobulin gene promoter. The TITF1 protein is encoded by a single gene in mice and humans. In mice, *Titf1* is located on chromosome 12, whereas in humans it is on chromosome 14q13. The distribution of the TITF1 protein and of the corresponding mRNA has been exhaustively studied in rodents [22]. TITF1 is expressed in the foregut and in the thyroid anlage during mammalian development and continues to be expressed in the thyroid follicular cells (TFC) in adulthood. TITF1 is also present in the trachea and lung bronchial epithelium and in selected areas of the forebrain, including the developing posterior pituitary. After birth and in adult animals, TITF1 is still present in the thyroid and lung epithelium and in the posterior pituitary, whereas its expression in the brain is restricted to periventricular regions and some hypothalamic nuclei. Gene inactivation experiments have revealed some important functions of TITF1 in vivo. The phenotype of mice homozygous for targeted disruption of the *Titf1* gene is rather complex, in accordance with the wide expression of this gene. *Titf1* null mice are characterized by impaired lung morphogenesis, lack of thyroid and pituitary glands, severe alterations in the ventral region of the forebrain, and death at birth [23]. Null mutation of *Titf1* also determines the failure of branching morphogenesis, resulting in PH [23,24]. Since *Titf1* is essential for lung development [25,26,27,28,29] and null mutation can cause PH in the mouse model, the authors undertook two lines of research to investigate the possible role of *TITF1* in the development of CDH in humans, namely: (a) an examination of *TITF1* expression in the trachea and lungs of human fetuses which died for causes other than CDH; and (b) the first direct *TITF1* mutation analysis of the genes of CDH patients.

## 2. Materials and Methods

### 2.1. Immunohistochemistry: Fetuses

We studied fetal foregut from 13 human fetuses without CDH at 14 to 24 weeks of gestation. All specimens were fixed in paraformaldehyde, embedded in paraffin and sectioned at 5 μm. Paraffin sections were dewaxed, rehydrated, and boiled by microwave oven (3 MW) with citrate buffer for 3 cycles of 5 min each. The sections were incubated overnight at 4 °C with anti-TITF1 primary antibody (1:100; clone 8G7G3/1; DakoCytomation). Staining was carried out with biotinylated secondary antibody (Biotinylated Link Universal) and streptavidin peroxidase conjugate (Streptavidin-HRP; Kit Lsab + System HRP, K0690; DakoCytomation), and DAB (Diaminobenzidine, K3466; DakoCytomation) as chromogen. Slides were counterstained with hematoxylin.

### 2.2. Molecular Genetics: CDH Patients

**Figure 1:**
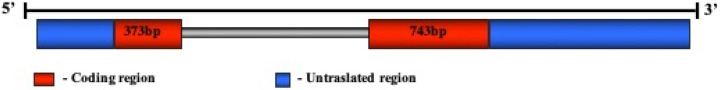
Exon II and III of *TITF1* gene targeted for genetic screening.

**Figure 2:**
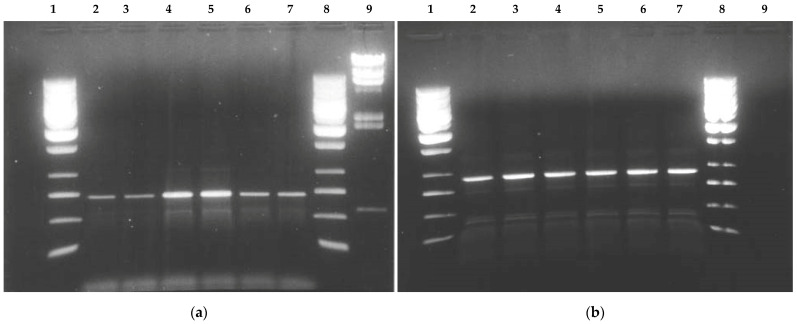
PCR amplification of exons, representative samples: (**a**) from left: lane 1 size molecular marker, lanes 2 to 7 samples amplification of the coding region exon II, lane 8 size molecular marker, lane 9 size and quantitative molecular marker; (**b**) from left: lane 1 size molecular marker, lanes 2 to 7 samples amplification of the coding region exon III, lane 8 size molecular marker.

Screening for mutations, insertions, and/or deletions was performed on genomic DNA from 16 patients (aged 2–7 days) affected by CDH (Table 1). Human *TITF1* is located on chromosome 14q13. We targeted exons II and III (Figure 1). DNA was extracted from frozen blood samples with Amersham Nucleon BAC3. We selected and synthesized two oligonucleotide pairs in the 5′ and 3′ external regions of the exon II and III, respectively, of the WT gene. We synthesized another set of nested oligos to obtain the complete exon sequences (Table 2). These oligonucleotides were used for PCR amplification of the two exons (Figure 2), and the resulting product was purified by gel electrophoresis. Sequences were performed in quadruplicate. Sequence reactions were purified automatically with a robotic station Biomek FX (Beckman Coulter, Brea, CA, USA) and obtained with a capillary electrophoresis sequencer 3730 DNA Analyzer (Applied Biosystems, Waltham, MA, USA). This procedure allowed us to obtain the entire sequences of the two exons in both strands, so each single nucleotide was sequenced several times in both directions. The sequences obtained were manually controlled and cleaned and then processed using bioinformatic tools to reconstruct the entire exon sequence like Blastn and Bioedit.

Finally, the reconstructed sequences of the two exons for each subject were aligned with the WT.

## 3. Results

### 3.1. Fetuses

**Figure 3:**
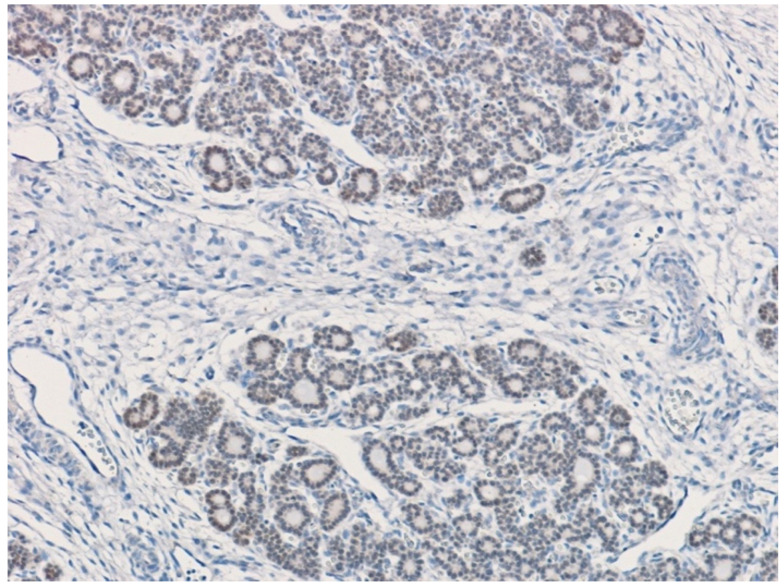
TITF1-positive nuclei (brown) of normal thyroid epithelium of a fetus at 14th week of gestation.

**Figure 4:**
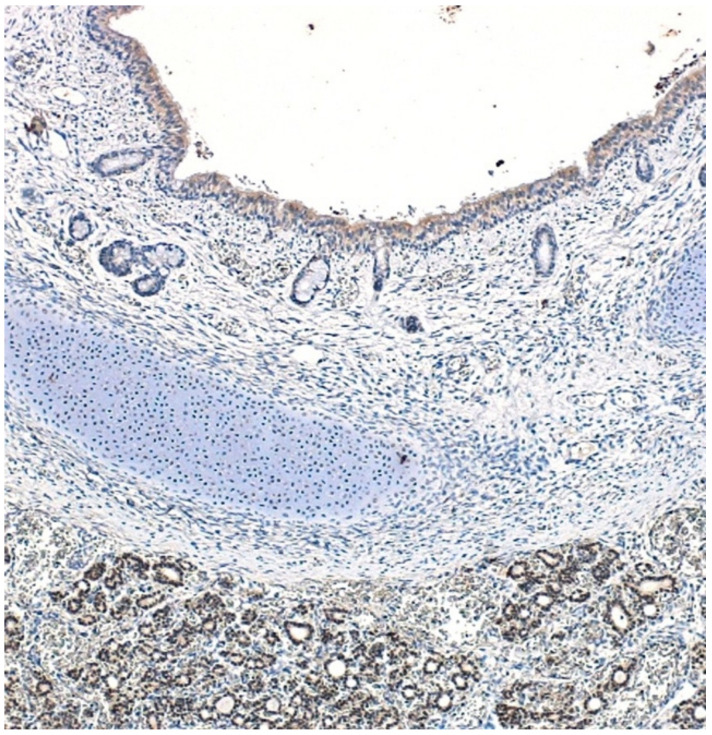
TITF1-positive nuclei (brown) are detected in both normal tracheal and thyroid. epithelium at 14th week of gestation.

We evaluated the distribution of TITF1 in 13 normal human fetuses at 14 to 24 weeks of gestation, spanning from early TITF1 expression in epithelial cells of human lung to its pronounced expression in epithelial cells of terminal airways [30]. Positive staining for TITF1 was found in follicular thyroid epithelium (Figure 3), tracheal epithelium (Figure 4), and lung bronchial epithelium of all subjects. At these gestational stages, the fetal esophageal wall was immununohistochemically negative.

### 3.2. CDH Patients

Comparison of *TITF1* from CDH patients with WT revealed no differences. No mutations, insertions, and/or deletions were detected.

## 4. Discussion

Although significant efforts have been made to explain the pathophysiology of CDH, our current understanding of the etiology remains unclear. CDH is a multifactorial and multigenic condition, and several genes have been identified and proposed as possible candidates. The Wnt pathway is required in diaphragm development [31], and accordingly in CDH patients, both copy number variations (CNVs) and single nucleotide variations (SNVs) have been detected in genes associated with it such as *WT1* [31,32] and *FZD2* [33]. There is evidence for a pivotal role of vitamin A signaling in the developing lung and diaphragm [34,35]. In CDH patients, CNVs of and SNVs in *STRA6*, which encodes a membrane receptor involved in the uptake of vitamin A [36], have been detected [37,38]. In CDH patients, CNVs have also been discovered in *ALDH1A2*, whose product is an enzyme that catalyzes the synthesis of retinoic acid from retinaldehyde, and in *RARA* and *RXRA*, which encode for retinoic acid receptor alpha and for Retinoid X Receptor Alpha, respectively, [39,40]. The region between 15q24 and 15q26 plays a critical role in the development of CDH [41]. *NR2F2* (*COUPTFII*), a member of a nuclear receptor superfamily, is highly expressed in the foregut [42] and resides within this minimal region. *NR2F2* may modulate the vitamin A pathway [43], and it is often deleted in CDH patients [41,44]. Consistent with this, *Nr2f2*-knockout mice develop diaphragmatic hernia [42]. In addition to mutations of genes associated with relevant signaling pathways, CNVs and SNVs have also been detected in other genes crucial during lung and diaphragm development. A list of candidate genes exists for CDH [45]. Because of the complexity of lung and diaphragm organogenesis and of CDH genetics, it is reasonable to consider the reported genes as predisposing to CDH rather than causative of CDH [45].

*TITF1* has long been considered a possible candidate gene in CDH pathogenesis because (i) it is expressed in lung endoderm and epithelium throughout lung development starting from 10 days of gestation, (ii) null mutation of *Titf1* in murine models determines a failure of tracheo-esophageal separation and branching resulting in PH [23,24], and (iii) the epithelial cells of these hypoplastic lungs do not undergo proper differentiation [23].

Our aim was to clarify whether *TITF1* gene plays a role in the complex etiology of CDH in humans. Although we confirmed the presence of TITF1 in follicular thyroid epithelium, normal trachea, and bronchial epithelium of human fetuses at 14 to 24 weeks of gestation, we did not detect any mutation, insertion, or deletion on the human *TITF1* gene in our series of CDH cases. Our results, represent the first direct mutation analysis of *TITF1* in a human CDH population. In conclusion, our screening showed no differences in *TITF1* sequencing between CDH patients and the WT gene. Our results do not support the hypothesis that *TITF1* mutations play a key role in the etiopathogenesis of CDH.

Interestingly, Chapin and associates [46] showed that in a rodent model of nitrofen- induced CDH, lung hypoplasia was associated with an increased expression of *Titf1*. They demonstrated that stimulation of lung growth through tracheal occlusion was followed by restoration of *Titf1* expression to levels comparable to non-hypoplastic lung and by lung maturation and weight increase. Their study suggests that misregulation of *Titf1* expression may be central in the disruption of branching morphogenesis and proximal–distal patterning in the lung. Further investigations are required to obtain additional information on genes (and their mechanisms of action) involved in CDH. This will impact the availability of fetal therapy and will allow to correlate genetic variants with clinical outcome for providing personalized counselling and therapies.

## Figures and Tables

**Table 1:** CDH patients.

	Male/Female	Sporadic/Familial	Disease during Pregnancy	Left/Right Broad	Pulmonary Hypoplasia	Associated Anomalies	Survivor
**CDH01**	M	Familial	-	Right	Severe	-	not
**CDH02**	M	Sporadic	-	Right	Severe	Pectus excavatum	yes
**CDH03**	F	Familial	-	Left	Mild		-
**CDH04**	M	Familial	-	Left	Mild	-	-
**CDH05**	F	Familial	-	Left	Mild	Tricuspid regurgitation	yes
**CDH06**	M	Sporadic	-	Left	Mild	-	yes
**CDH07**	F	Sporadic	-	Left	Mild	-	yes
**CDH08**	F	Sporadic	-	Left	Mild	-	yes
**CDH09**	F	Sporadic	-	Left	Mild	-	yes
**CDH10**	M	Sporadic	-	Left	Mild		
**CDH11**	M	Sporadic	-	Right	Mild	-	yes
**CDH12**	F	Sporadic	-	Left	Mild	-	-
**CDH13**	M	Sporadic	-	Left	Mild	-	yes
**CDH14**	M	Sporadic	-	Left	Mild		yes
**CDH15**	F	Sporadic	-	Right	Severe	Pectus excavatum	yes
**CDH16**	M	Sporadic	-	Left	Mild		yes

**Table 2:** Oligonucleotides for PCR and/or sequencing.

Name	Type	Sequence 5′→3′
hTiTF1 exIIF	Exon II PCR/Seq Forward	TGG CTG CCT AAA ACC TG
hTiTF1 exIIR	Exon II PCR/Seq Reverse	GCC CTC CCT GAT GC
hTiTF1 exIIF2	Exon II Seq Forward	GGA AAG CTA CAA GAA AGT GGG
hTiTF1 exIIR2	Exon II Seq Reverse	CTG TTC CTC ATG GTG TCC TGG
hTiTF1 exIIPCRUP	Exon II PCR/Seq Forward	GAG GAC TCG GTC CAC TCC GTT AC
hTiTF1 exIIPCRDw	Exon II PCR/Seq Reverse	AGC GCT ACC AAG TGC CTG TTC TTG
hTiTF1 exIIIF	Exon III PCR/Seq Forward	AGG GTT GGG GCT GTG AG
hTiTF1 exIIIR	Exon III PCR/Seq Reverse	GGA TGG TCT GTG TGG
hTiTF1 exIIIF2	Exon III Seq Forward	ATG GCG CGG AAA ACA GG
hTiTF1 exIIIR2	Exon III Seq Reverse	GCG GTG GAT GGT CA
hTiTF1 exIIIF3	Exon III Seq Forward	GCT TCA AGC AAC AGA AGT ACC
hTiTF1 exIIIR3	Exon III Seq Reverse	ACG GTT TGC CGT CTT TCA CC
hTiTF1 exIIIF4	Exon III Seq Forward	AAC AGG CTC AGC AGT CG
hTiTF1 exIIIR4	Exon III Seq Reverse	GTC AGG TGG ATC ATG CTG G
